# Free-living marine nematodes from San Julián Bay (Santa Cruz, Argentina)

**DOI:** 10.3897/zookeys.489.7311

**Published:** 2015-03-24

**Authors:** Catalina Pastor de Ward, Virginia Lo Russo, Gabriela Villares, Viviana Milano, Lidia Miyashiro, Renato Mazzanti

**Affiliations:** 1 Laboratorio de Meiobentos CENPAT-CONICET, Boulevard Brown 2915, U9120ACF, Puerto Madryn, Argentina; 2 Universidad Nacional de la Patagonia San Juan Bosco, sede Puerto Madryn. Boulevard Brown 3051, U9120ACF, Puerto Madryn, Argentina; 3 Centro de Cómputos CENPAT-CONICET, Boulevard Brown 2915, U9120ACF, Puerto Madryn, Argentina

**Keywords:** Nematoda, Enoplea, Chromadorea, South Atlantic

## Abstract

The free-living marine nematodes of San Julián Bay dataset is based on sediment samples collected in January 2009 during the project PICT AGENCIA-FONCYT 2/33345-2005. A total of 36 samples have been taken at three locations in the San Julián Bay, Santa Cruz Province, Argentina on the coastal littoral at three tidal levels. This presents a unique and important collection for the nematode benthic biodiversity assessment as this area remains one of the least known regions in Patagonia. In total 10,030 specimens of free-living marine nematodes belonging to 2 classes, 9 orders, 35 families, 78 genera and 125 species were collected. The San Julián city site presented a very high species richness.

## Data published through

GBIF: http://www.gbif.org/dataset/06df03fc-8973-490c-af74-089fffae9e24

## Taxonomic coverage description

**Figure 1.**
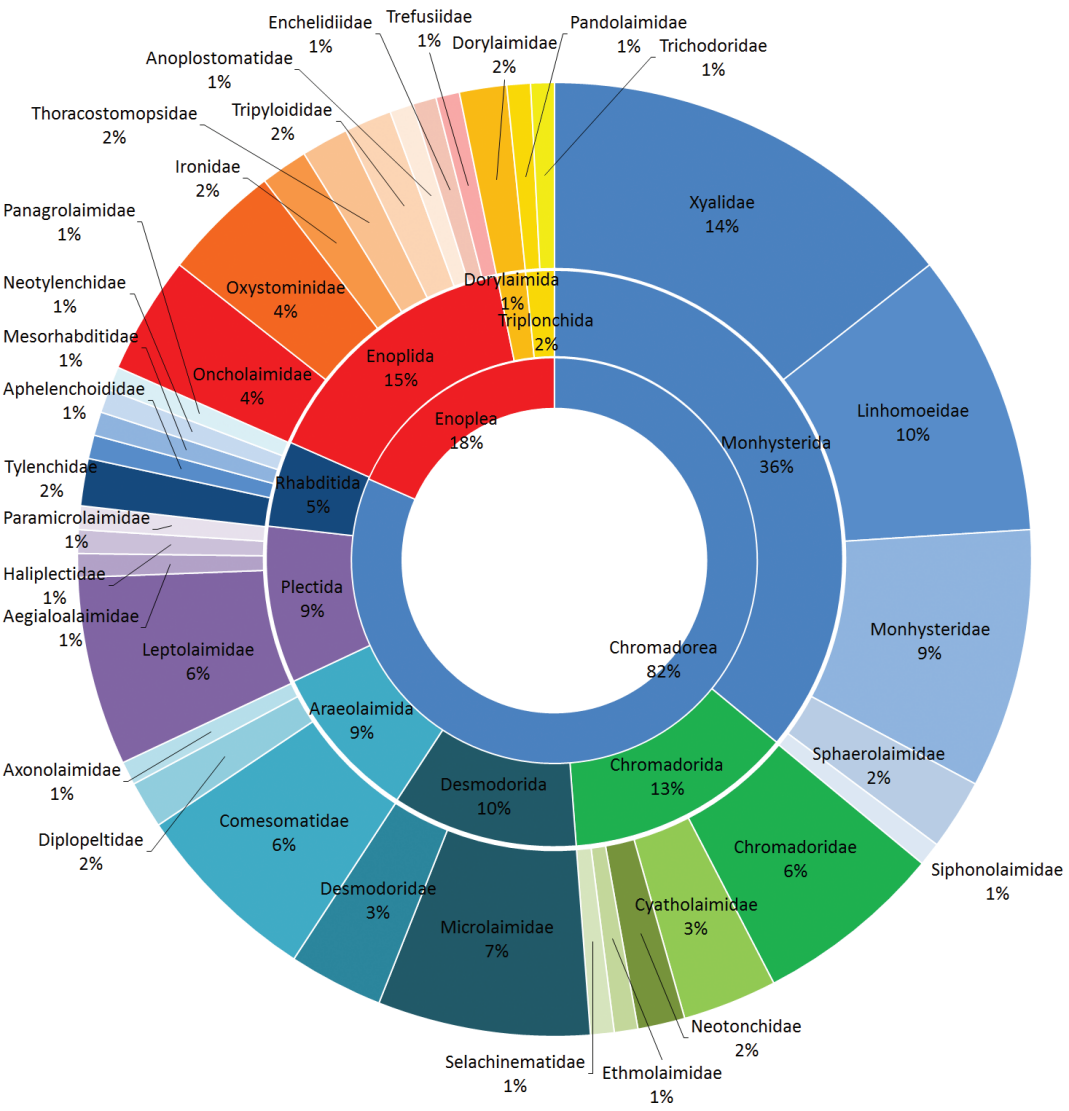
Taxonomic coverage by class, order and family.

This is the first study on nematodes performed on a sub-Antarctic salt marsh along the coast of Santa Cruz Province, Argentina with a growing human impact (oil ventures, mining, aquaculture and tourism). The objectives of the study were to collect, identify and discover the structure and diversity of nematode community of San Julián Bay. The coverage (Figure 1) of this dataset includes two classes: Chromadorea (82%) and Enoplea (18%); nine orders: with Monhysterida (36%), followed by Enoplida (15%) and Chromadorida (13%) as those of main occurrences and thirty-five families (see Figure 1).

## Taxonomic ranks

**Kingdom:** Animalia

**Phylum:** Nematoda

**Class:** Chromadorea, Enoplea

**Order:** Monhysterida, Enoplida, Chromadorida, Desmodorida, Araeolaimida, Plectida, Rhabditida, Dorylaimida, Triplonchida

**Family:** Xyalidae, Linhomoeidae, Monhysteridae, Microlaimidae, Chromadoridae, Comesomatidae, Leptolaimidae, Oncholaimidae, Oxystominidae, Cyatholaimidae, Desmodoridae, Sphaerolaimidae, Diplopeltidae, Dorylaimidae, Ironidae, Neotonchidae, Thoracostomopsidae, Tripyloididae, Tylenchidae, Aegialoalaimidae, Anoplostomatidae, Aphelenchoididae, Axonolaimidae, Enchelidiidae, Ethmolaimidae.

**Genera:** *Odontophora*, *Hopperia*, *Laimella*, *Sabatieria*, *Campylaimus*, *Chromadora*, *Chromadorella*, *Prochromadora*, *Dichromadora*, *Neochromadora*, *Spilophorella*, *Marylynnia*, *Paracanthonchus*, *Paracyatholaimus*, *Pomponema*, *Paraethmolaimus*, *Gomphionema*, *Neotonchus*, *Halichoanolaimus*, *Molgolaimus*, *Polysigma*, *Spirinia*, *Bolbolaimus*, *Microlaimus*, *Desmolaimus*, *Metalinhomoeus*, *Terschellingia*, *Paralinhomoeus*, *Siphonolaimus*, *Diplolaimella*, *Diplolaimelloides*, *Halomonhystera*, *Monhystera*, *Sphaerolaimus*, *Subsphaerolaimus*, *Amphimonhystera*, *Daptonema*, *Linhystera*, *Metadesmolaimus*, *Paramonohystera*, *Pseudosteineria*, *Steineria*, *Theristus*, *Haliplectus*, *Cyartonema*, *Camacolaimus*, *Deontolaimus*, *Antomicron*, *Leptolaimoides*, *Leptolaimus*, *Paramicrolaimus*, *Mesorhabditis*, *Aphelenchoides*, *Panagrolaimus*, *Boleodorus*, *Tylenchorhynchus*, *Tylenchus*, *Dorylaimus*, *Eudorylaimus*, *Chaetonema*, *Thoracostomopsis*, *Dolicholaimus*, *Syringolaimus*, *Halalaimus*, *Thalassoalaimus*, *Wieseria*, *Calyptronema*, *Adoncholaimus*, *Oncholaimellus*, *Viscosia*, *Oncholaimus*, *Rhabdocoma*, *Bathylaimus*, *Tripyloides*, *Trichodorus*, *Pandolaimus*.

**Species with higher occurrences:** *Paraethmolaimus dahli*, *Sabatieria mortenseni*, *Daptonema rectangulatum*, *Metalinhomoeus parafiliformis*, *Leptolaimus puccinelliae*, *Diplolaimelloides oschei*, *Leptolaimus sebastiani*, *Metalinhomoeus gloriae*, *Thalassomonhystera parva*, *Metalinhomoeus typicus*, *Haliplectus salicornius*.

## Spatial coverage

**Figure 2.**
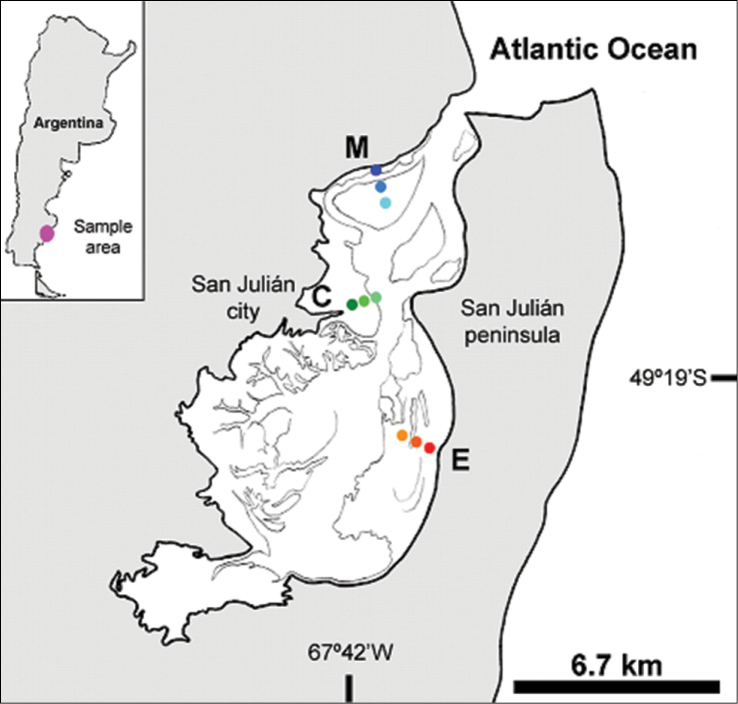
Spatial coverage. San Julián Bay, Argentina. Sites: M = “La pingüinera”, C = “La Rural”, E = “El Rincón”. Levels = u, m, i.

**Figure 3.**
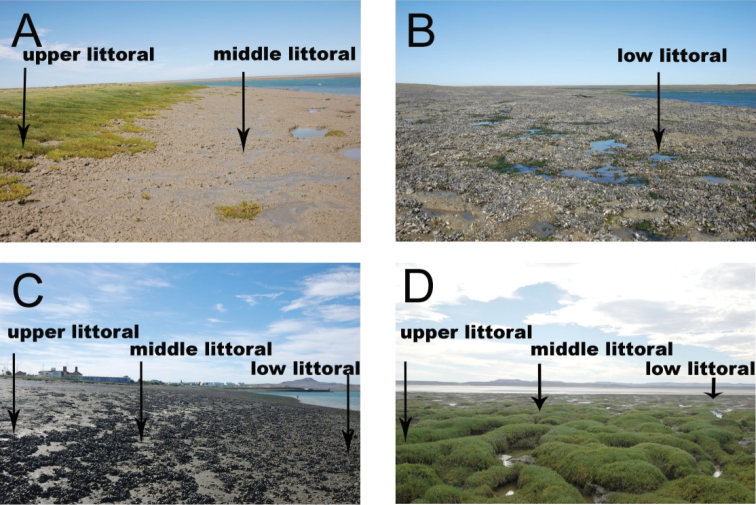
San Julián Bay, Argentina. Views from the sampling sites. **A, B** “La pingüinera” (M) **C** “La Rural”(C) **D** “El Rincón” (D).

**General spatial coverage:** San Julián Bay, Santa Cruz Province, Argentina (Figure 2). For this study three sites were selected: “La pingüinera”(M), at the bay entrance, “La Rural” (C) in front of San Julián city and “El Rincón” (E) at the end of the bay. At each sampling site, three tidal levels were chosen: upper-littoral, high tide, salt-marsh habitat (u); middle littoral, mean tide, un-vegetated habitat (m) and low littoral, low tide, un-vegetated habitat (l) (Figure 3).

**Coordinates:** La pingüinera: Mu = 49°16'15.24"S; 67°42'40.68"W; Mm = 49°16'12"S; 67°42'43.92"W; Ml = 49°16'11.28"S; 67°42'39.6"W. La Rural: Cu = 49°18'37.44"S; 67°42'55.8"W; Cm = 49°18'34.92"S; 67°42'55.8"W; Cl = 49°18'35.28"S; 67°42'52.56"W. El Rincón: Eu = 49°21'18.72"S; 67°41'26.88"W; Em = 49°21'14.4"S; 67°41'42.36"W; El = 49°21'18"S; 67°41'51"W.

## Temporal coverage

11–13 January 2009.

## Methods

**Sampling description:** At each site and level location, four replicates (20 ml) were sampled with a PVC syringe (60 ml, inner diameter 2.9 cm) and separated by a distance of 5-10 m each: four for marine nematodes counts, two for organic matter and two for sediment analyses. Each sample was fixed *in situ*, with a solution of 5% formaldehyde in filtered sea water with the addition of Rose Bengal tint.

Marine nematodes were extracted from samples using the elutriation/decantation LUDOX TM (colloidal silica polymer) method at a specific gravity of 1.15, quantifying only organisms passing through a 500 µm mesh and then retained by a 63 µm mesh. Samples were evaporated to anhydrous glycerol and permanent slides made (Somerfield and Warwick 1996).

The taxonomic classification followed proposed by De Ley and Blaxter (2004). For the identification of species international keys (Platt and Warwick 1983, Platt and Warwick 1988, Warwick et al. 1998, Lorenzen 1994, Abebe et al. 2006) and previous taxonomical papers for Santa Cruz nematodes (Pastor de Ward 1978, 1980, 1984a, b, c, d, e, 1985, 1986, 1988, 1989, 1990, 1991, 1993, 1994, 1995a, b, 1996, 1998a, b, c, 1999, Pastor de Ward and Lo Russo 2009, Villares and Pastor de Ward 2012, Villares et al. 2013, Pastor de Ward et al. 2013) were used.

## Project details

**Project title:** “*Evaluación del impacto urbano en costas areno-limosas de la provincia de Santa Cruz, usando métodos rápidos de análisis de cambios en estructura comunitaria del bentos*.” [Impact assessment in urban sand-clay coastal areas of Santa Cruz Province, using methods of rapid assessment in changes of nematodes community structure].

**Personnel:** Catalina Pastor de Ward (Project Director, meio-benthos specialist); Héctor Zaixso (Project Co-director, macro-benthos specialist), Virginia Lo Russo (Field work, nematodes identification, data collection and analysis), Gabriela Villares (Data collection and analysis), Viviana Milano (Grant student, data input), Lidia Miyashiro (Darwin core data input), Renato Mazzanti (Software engineer, data base manager).

**Funding:** PICT AGENCIA-FONCYT 2/33345-2005

**Study extent description:** The San Julián Bay marine nematodes is a dataset that gives new insights on the taxonomic and geographic distribution of south Atlantic marine nematodes, covering an under-explored region of the southern Atlantic coasts. This is the first study on marine nematodes in this locality. This dataset presents species occurrences and species richness of the individual free-living marine nematodes present at three coastal areas (La pingüinera; La Rural; El Rincón) of the San Julián Bay at three different tidal levels (upper, middle and low-littoral).

In total 10,030 specimens of free-living marine nematodes belonging to 2 classes, 9 orders, 35 families, 78 genera and 125 species were collected.

**Table 1.** Collected species.

Genera and species	Family	Order	Class
*Odontophora peritricha* Wieser, 1956	Axonolaimidae	Araeolaimida	Chromadorea
*Hopperia americana* Pastor de Ward, 1984	Comesomatidae	Araeolaimida	Chromadorea
*Hopperia arntzi* Chen & Vincx, 1998	Comesomatidae	Araeolaimida	Chromadorea
*Laimella* sp. 1	Comesomatidae	Araeolaimida	Chromadorea
*Laimella* sp. 2	Comesomatidae	Araeolaimida	Chromadorea
*Sabatieria* sp. 1	Comesomatidae	Araeolaimida	Chromadorea
*Sabatieria* sp. 2	Comesomatidae	Araeolaimida	Chromadorea
*Sabatieria mortenseni* (Ditlevsen, 1921)	Comesomatidae	Araeolaimida	Chromadorea
*Sabatieria wieseri* Platt, 1985	Comesomatidae	Araeolaimida	Chromadorea
*Campylaimus gerlachi* Timm, 1961	Diplopeltidae	Araeolaimida	Chromadorea
*Campylaimus* sp. 1	Diplopeltidae	Araeolaimida	Chromadorea
*Chromadora nudicapitata* Bastian, 1865	Chromadoridae	Chromadorida	Chromadorea
*Chromadorella circumflexa* Wieser, 1954	Chromadoridae	Chromadorida	Chromadorea
*Prochromadora argentinensis* Pastor de Ward, 1984	Chromadoridae	Chromadorida	Chromadorea
*Dichromadora* sp. 1	Chromadoridae	Chromadorida	Chromadorea
*Neochromadora lineata* Pastor de Ward, 1985	Chromadoridae	Chromadorida	Chromadorea
*Neochromadora papillosa* Pastor de Ward, 1985	Chromadoridae	Chromadorida	Chromadorea
*Neochromadora* sp. 1	Chromadoridae	Chromadorida	Chromadorea
*Spilophorella paradoxa* (De Man, 1888)	Chromadoridae	Chromadorida	Chromadorea
*Marylynnia quadriseta* (Wieser, 1954)	Cyatholaimidae	Chromadorida	Chromadorea
*Paracanthonchus longispiculum* Pastor de Ward, 1985	Cyatholaimidae	Chromadorida	Chromadorea
*Paracyatholaimus chilensis* Gerlach, 1953	Cyatholaimidae	Chromadorida	Chromadorea
*Pomponema tautraense* (Allgén, 1933)	Cyatholaimidae	Chromadorida	Chromadorea
*Paraethmolaimus dahli* (Gerlach, 1953)	Ethmolaimidae	Chromadorida	Chromadorea
*Gomphionema* sp. 1	Neotonchidae	Chromadorida	Chromadorea
*Neotonchus* sp. 1	Neotonchidae	Chromadorida	Chromadorea
*Halichoanolaimus ovalis* Ditlevsen, 1921	Selachinematidae	Chromadorida	Chromadorea
*Molgolaimus minutus* Jensen, 1978	Desmodoridae	Desmodorida	Chromadorea
*Molgolaimus* sp. 1	Desmodoridae	Desmodorida	Chromadorea
*Polysigma* sp. 1	Desmodoridae	Desmodorida	Chromadorea
*Spirinia septentrionalis* (Cobb, 1914)	Desmodoridae	Desmodorida	Chromadorea
*Bolbolaimus* sp. 1	Microlaimidae	Desmodorida	Chromadorea
*Bolbolaimus* sp. 3	Microlaimidae	Desmodorida	Chromadorea
*Microlaimus capillaris* Gerlach, 1957	Microlaimidae	Desmodorida	Chromadorea
*Microlaimus conothelis* (Lorenzen, 1973)	Microlaimidae	Desmodorida	Chromadorea
*Microlaimus cyatholaimoides* Gerlach, 1957	Microlaimidae	Desmodorida	Chromadorea
*Microlaimus decoratus* Pastor de Ward, 1991	Microlaimidae	Desmodorida	Chromadorea
*Microlaimus gerlachi* Wieser, 1954	Microlaimidae	Desmodorida	Chromadorea
*Microlaimus globiceps* De Man, 1880	Microlaimidae	Desmodorida	Chromadorea
*Microlaimus* sp. 1	Microlaimidae	Desmodorida	Chromadorea
*Desmolaimus* sp. 1	Linhomoeidae	Monhysterida	Chromadorea
*Desmolaimus* sp. 2	Linhomoeidae	Monhysterida	Chromadorea
*Metalinhomoeus gloriae* Pastor de Ward, 1989	Linhomoeidae	Monhysterida	Chromadorea
*Metalinhomoeus parafiliformis* Pastor de Ward, 1989	Linhomoeidae	Monhysterida	Chromadorea
*Metalinhomoeus typicus* De Man, 1907	Linhomoeidae	Monhysterida	Chromadorea
*Terschellingia distalamphida* Juario, 1974	Linhomoeidae	Monhysterida	Chromadorea
*Terschellingia longicaudata* De Man, 1907	Linhomoeidae	Monhysterida	Chromadorea
*Terschellingia* sp. 1	Linhomoeidae	Monhysterida	Chromadorea
*Terschellingia sulfidrica* Pastor de Ward, 1989	Linhomoeidae	Monhysterida	Chromadorea
*Paralinhomoeus aridus* Pastor de Ward, 1989	Linhomoeidae	Monhysterida	Chromadorea
*Paralinhomoeus pachyamphis* Wieser, 1956	Linhomoeidae	Monhysterida	Chromadorea
*Paralinhomoeus visitus* Pastor de Ward, 1989	Linhomoeidae	Monhysterida	Chromadorea
*Siphonolaimus auratus* Wieser, 1956	Siphonolaimidae	Monhysterida	Chromadorea
*Diplolaimella gerlachi* Pastor de Ward, 1984	Monhysteridae	Monhysterida	Chromadorea
*Diplolaimelloides oschei* Meyl, 1954	Monhysteridae	Monhysterida	Chromadorea
*Diplolaimelloides tehuelchus* Pastor de Ward & Lo Russo, 2009	Monhysteridae	Monhysterida	Chromadorea
*Diplolaimelloides warwicki* Pastor de Ward & Lo Russo, 2009	Monhysteridae	Monhysterida	Chromadorea
*Halomonhystera disjuncta* (Bastian, 1865)	Monhysteridae	Monhysterida	Chromadorea
*Halomonhystera* sp. 1	Monhysteridae	Monhysterida	Chromadorea
*Halomonhystera* sp. 2	Monhysteridae	Monhysterida	Chromadorea
*Halomonhystera* sp. 3	Monhysteridae	Monhysterida	Chromadorea
*Thalassomonhystera parva* (Bastian, 1865)	Monhysteridae	Monhysterida	Chromadorea
*Thalassomonhystera refringens* (Bresslau & Stekhoven, 1935)	Monhysteridae	Monhysterida	Chromadorea
*Sphaerolaimus pacificus* Allgen 1947	Sphaerolaimidae	Monhysterida	Chromadorea
*Sphaerolaimus pentasetus* Pastor de Ward, 1984	Sphaerolaimidae	Monhysterida	Chromadorea
*Subsphaerolaimus* sp. 1	Sphaerolaimidae	Monhysterida	Chromadorea
*Amphimonhystera* sp. 1	Xyalidae	Monhysterida	Chromadorea
*Daptonema concordiense* Pastor de Ward, 1985	Xyalidae	Monhysterida	Chromadorea
*Daptonema laxus* Wieser, 1956	Xyalidae	Monhysterida	Chromadorea
*Daptonema lopezi* Pastor de Ward, 1985	Xyalidae	Monhysterida	Chromadorea
*Daptonema rectangulatum* Pastor de Ward, 1985	Xyalidae	Monhysterida	Chromadorea
*Daptonema* sp. 1	Xyalidae	Monhysterida	Chromadorea
*Linhystera longa* Pastor de Ward, 1985	Xyalidae	Monhysterida	Chromadorea
*Metadesmolaimus* sp. 1	Xyalidae	Monhysterida	Chromadorea
*Metadesmolaimus* sp. 2	Xyalidae	Monhysterida	Chromadorea
*Paramonohystera megacephala* (Steiner, 1916)	Xyalidae	Monhysterida	Chromadorea
*Paramonohystera parabutschlii* Timm, 1961	Xyalidae	Monhysterida	Chromadorea
*Paramonohystera* sp. 1	Xyalidae	Monhysterida	Chromadorea
*Paramonohystera* sp. 2	Xyalidae	Monhysterida	Chromadorea
*Paramonohystera* sp. 3	Xyalidae	Monhysterida	Chromadorea
*Pseudosteineria anticipans* Wieser, 1956	Xyalidae	Monhysterida	Chromadorea
*Steineria pilosa* Cobb, 1914	Xyalidae	Monhysterida	Chromadorea
*Theristus modicus* Wieser, 1956	Xyalidae	Monhysterida	Chromadorea
*Theristus* sp. 1	Xyalidae	Monhysterida	Chromadorea
*Haliplectus salicornius* Pastor de Ward, 1984	Haliplectidae	Plectida	Chromadorea
*Cyartonema flexile* Cobb, 1920	Aegialoalaimidae	Plectida	Chromadorea
*Camacolaimus barbatus* Warwick, 1970	Leptolaimidae	Plectida	Chromadorea
*Deontolaimus papillatus* De Man, 1880	Leptolaimidae	Plectida	Chromadorea
*Antomicron alveolatum* Villares & Pastor de Ward, 2012	Leptolaimidae	Plectida	Chromadorea
*Leptolaimoides* sp. 1	Leptolaimidae	Plectida	Chromadorea
*Leptolaimoides* sp. 2	Leptolaimidae	Plectida	Chromadorea
*Leptolaimus gabinoi* Villares & Pastor de Ward, 2012	Leptolaimidae	Plectida	Chromadorea
*Leptolaimus puccinelliae* Gerlach, 1959	Leptolaimidae	Plectida	Chromadorea
*Leptolaimus sebastiani* Vitiello, 1974	Leptolaimidae	Plectida	Chromadorea
*Paramicrolaimus spirulifer* Wieser, 1959	Paramicrolaimidae	Plectida	Chromadorea
*Mesorhabditis* sp. 1	Mesorhabditidae	Rhabditida	Chromadorea
*Aphelenchoides* sp. 1	Aphelenchoididae	Rhabditida	Chromadorea
*Panagrolaimus* sp. 1	Panagrolaimidae	Rhabditida	Chromadorea
*Boleodorus* sp. 1	Neotylenchidae	Rhabditida	Chromadorea
*Tylenchorhynchus* sp. 1	Tylenchidae	Rhabditida	Chromadorea
*Tylenchus* sp. 1	Tylenchidae	Rhabditida	Chromadorea
*Dorylaimus* sp. 1	Dorylaimidae	Dorylaimida	Enoplea
*Eudorylaimus* sp. 1	Dorylaimidae	Dorylaimida	Enoplea
*Chaetonema* sp. 1	Anoplostomatidae	Enoplida	Enoplea
*Thoracostomopsis* sp. 1	Thoracostomopsidae	Enoplida	Enoplea
*Dolicholaimus marioni* De Man, 1888	Ironidae	Enoplida	Enoplea
*Syringolaimus smarigdus* Cobb, 1928	Ironidae	Enoplida	Enoplea
Halalaimus (Halalaimus) setosus Timm, 1961	Oxystominidae	Enoplida	Enoplea
Halalaimus (Nuada) diacros Mawson, 1958	Oxystominidae	Enoplida	Enoplea
*Halalaimus* sp. 3	Oxystominidae	Enoplida	Enoplea
*Halalaimus floridanus* Keppner, 1992	Oxystominidae	Enoplida	Enoplea
*Thalassoalaimus* sp. 1	Oxystominidae	Enoplida	Enoplea
*Wieseria* sp. 1	Oxystominidae	Enoplida	Enoplea
*Calyptronema maxweberi* (De Man, 1922)	Enchelidiidae	Enoplida	Enoplea
*Eurystomina* sp. 1	Enchelidiidae	Enoplida	Enoplea
*Adoncholaimus* sp. 1	Enchelidiidae	Enoplida	Enoplea
*Oncholaimellus paracarlbergi* Pastor de Ward, 1993	Oncholaimidae	Enoplida	Enoplea
*Viscosia macramphida* Chitwood, 1951	Oncholaimidae	Enoplida	Enoplea
*Viscosia separabilis* (Wieser, 1953)	Oncholaimidae	Enoplida	Enoplea
*Oncholaimus salobrus* Pastor de Ward, 1993	Oncholaimidae	Enoplida	Enoplea
*Rhabdocoma* sp. 1	Trefusiidae	Enoplida	Enoplea
*Bathylaimus australis* Cobb, 1894	Tripyloididae	Enoplida	Enoplea
*Tripyloides amazonicus* (Gerlach, 1957)	Tripyloididae	Enoplida	Enoplea
*Trichodorus* sp. 1	Trichodoridae	Triplonchida	Enoplea
*Pandolaimus* sp. 1	Pandolaimidae	Triplonchida	Enoplea

**Quality control description:** The geo-referencing of all specimens were recorded using a Garmin eTrex Legend GPS (WGS84 Datum) with an accuracy of less than 10 m and with at least 5 satellites.

The taxonomic identification of specimens, scientific names, and their current accurate spelling were verified by C. Pastor de Ward, a free-living marine nematode specialist. Other post-validation procedures (including geographic coordinate format, congruence between collection and identification dates, absence of ASCII anomalous characters) were checked using the Darwin Test software (http://www.gbif.es/darwin_test/Darwin_Test_in.php).

## Dataset description

**Object name:** Darwin Core Archive free-living marine Nematodes from San Julián Bay (Santa Cruz, Argentina)

**Character encoding:** UTF-8

**Format name:** Darwin Core Archive format

**Format version:** 1.0

**Distribution:** http://www.cenpat-conicet.gov.ar:8080/ipt-2.0.3/resource.do?r=sjnem

**Publication date of data:** 2013-10-17

**Language:** English

**Licenses of use:** This work is licensed under a Creative Commons CC0 1.0 License http://creativecommons.org/publicdomain/zero/1.0/legalcode

## External datasets

**Object name:** Centro Nacional Patagónico (CENPAT-CONICET)

**Distribution:** http://www.cenpat-conicet.gov.ar:8080/ipt-2.0.3/resource.do?r=sjnem

**Object name:** Ministerio de Ciencia y Tecnología de Argentina (Sistema Nacional de Datos Biológicos - SNDB)

**Distribution:** GBIF: http://www.gbif.org/dataset/06df03fc-8973-490c-af74-089fffae9e24

**Formatted:** English (U.K.)

**Field Code Changed**

**Metadata language:** English

**Date of metadata creation:** 2013-10-17

**Hierarchy level:** Dataset
